# Meta gene set enrichment analyses link miR-137-regulated pathways with schizophrenia risk

**DOI:** 10.3389/fgene.2015.00147

**Published:** 2015-04-20

**Authors:** Carrie Wright, Vince D. Calhoun, Stefan Ehrlich, Lei Wang, Jessica A. Turner, Nora I. Perrone- Bizzozero

**Affiliations:** ^1^The Mind Research NetworkAlbuquerque, NM, USA; ^2^Department of Neurosciences, University of New MexicoAlbuquerque, NM, USA; ^3^Department of Electrical and Computer Engineering, University of New MexicoAlbuquerque, NM, USA; ^4^Translational Developmental Neuroscience Section, Department of Child and Adolescent Psychiatry, Faculty of Medicine, Technische Universität DresdenDresden, Germany; ^5^Department of Psychiatry, Harvard Medical School, Massachusetts General HospitalBoston, MA, USA; ^6^Athinoula A. Martinos Center for Biomedical Imaging, Massachusetts General Hospital/Massachusetts Institute of Technology/Harvard Medical SchoolCharlestown, MA, USA; ^7^Department of Psychiatry and Behavioral Sciences, Northwestern University Feinberg School of MedicineChicago, IL, USA; ^8^Department of Radiology, Northwestern University Feinberg School of MedicineChicago, IL, USA; ^9^Department of Psychology and Neuroscience Institute, Georgia State UniversityAtlanta, GA, USA; ^10^Department of Psychiatry, University of New MexicoAlbuquerque, NM, USA

**Keywords:** schizophrenia, miR-137, microRNA, gene set enrichment analysis (GSEA), pathway analysis, PKA signaling

## Abstract

**Background**: A single nucleotide polymorphism (SNP) within *MIR137*, the host gene for miR-137, has been identified repeatedly as a risk factor for schizophrenia. Previous genetic pathway analyses suggest that potential targets of this microRNA (miRNA) are also highly enriched in schizophrenia-relevant biological pathways, including those involved in nervous system development and function.

**Methods**: In this study, we evaluated the schizophrenia risk of miR-137 target genes within these pathways. Gene set enrichment analysis of pathway-specific miR-137 targets was performed using the stage 1 (21,856 subjects) schizophrenia genome wide association study data from the Psychiatric Genomics Consortium and a small independent replication cohort (244 subjects) from the Mind Clinical Imaging Consortium and Northwestern University.

**Results**: Gene sets of potential miR-137 targets were enriched with variants associated with schizophrenia risk, including target sets involved in axonal guidance signaling, Ephrin receptor signaling, long-term potentiation, PKA signaling, and Sertoli cell junction signaling. The schizophrenia-risk association of SNPs in PKA signaling targets was replicated in the second independent cohort.

**Conclusions**: These results suggest that these biological pathways may be involved in the mechanisms by which this *MIR137* variant enhances schizophrenia risk. SNPs in targets and the miRNA host gene may collectively lead to dysregulation of target expression and aberrant functioning of such implicated pathways. Pathway-guided gene set enrichment analyses should be useful in evaluating the impact of other miRNAs and target genes in different diseases.

## Introduction

MicroRNAs (miRNAs) are a class of noncoding RNAs involved in posttranscriptional gene expression regulation by binding to complementary sequences within target mRNAs (Bartel, 2009). miRNAs play a role in a variety of cellular processes and diseases (Henrion-Caude et al., 2012), including psychiatric disorders (Mellios and Sur, 2012). Several lines of evidence support a role of miR-137 in schizophrenia, a severe mental illness characterized by symptoms of delusions, hallucinations, and diminished sociability. Not only was the strongest associated SNP identified in the first large schizophrenia genome wide association study (GWAS) located in the host gene of miR-137 (Ripke et al., 2011) but also the other top four polymorphisms mapped to validated target genes of this miRNA (Kwon et al., 2013). This miRNA is involved in several steps in neuronal development, from regulation of neuronal proliferation and differentiation (Silber et al., 2008; Smrt et al., 2010; Szulwach et al., 2010; Sun et al., 2011) to dendritic arborization (Smrt et al., 2010), suggesting that the risk allele may impact these processes. Additionally, imaging genetics studies have found distinct alterations associated with the *MIR137* risk variant in subjects with schizophrenia. One recent functional magnetic resonance imaging (fMRI) study identified alterations in brain activity patterns during a sentence completion task within the amygdala and pre and postcentral gyrus only in risk allele carrier subjects that were also at risk for schizophrenia development (Whalley et al., 2012). Structural imaging analyses also identified patient-specific alterations in risk allele carriers such as reduced whole brain functional anisotropy, reduced left hippocampal volume and enlarged right and left lateral ventricle volume (Lett et al., 2013). In contrast, no volumetric alterations were found in protective-allele carrying patients or healthy risk allele carriers (Li and Su, 2013). The disease-specific risk allele associations found in these studies suggest that, in agreement with the evidence of polygenic risk in schizophrenia (Purcell et al., 2009), other genetic factors besides the *MIR137* SNP may underlie disease-specific abnormalities in brain structure and function. Polymorphisms within multiple miR-137 targets may in part increase genetic risk by potentially enhancing dysregulation by this miRNA. Variants within or adjacent to miRNA recognition sites in 3′ UTRs can alter binding and binding availability of miRNAs to target mRNAs, leading to altered gene expression and phenotypic or disease states (Abelson, 2005; Wang et al., 2008) Therefore, collective polymorphisms within miR-137 target genes in schizophrenia-relevant pathways may disrupt regulation by this miRNA, and/or lead to a general disruption of the pathways in the patients.

Initial bioinformatics analyses of the function of putative and validated targets suggest that miR-137 target genes are involved in many schizophrenia relevant pathways, including axonal guidance signaling, Ephrin receptor signaling, synaptic long term potentiation (LTP), and protein kinase A (PKA) signaling, among others (Wright et al., 2013). Besides LTP, little is known about the role of these miR-137 regulated pathways in schizophrenia. Preliminary SNP by SNP association analyses performed by the PGC found significant enrichment of risk associated SNPs within a subset of predicted miR-137 target genes (Ripke et al., 2011) and this was replicated with a larger set of predicted targets, using a joint gene set enrichment analysis (Ripke et al., 2013, 2014). However, no studies to date have examined the collective risk of miR-137 target SNPs across biological pathways.

The goals of this study were to assess the schizophrenia-risk of both experimentally validated and high confidence predicted miR-137 targets, and to evaluate for the first time the risk association of these targets in a pathway-specific manner. These analyses were performed using meta gene set enrichment analyses of specific target gene sets (Segrè et al., 2010). Gene set enrichment analysis (GSEA) is particularly useful in the case of polygenic diseases such as schizophrenia (Purcell et al., 2009) as it allows for examination of the collective effect of multiple polymorphisms. Furthermore, analysis of gene sets in a pathway specific framework can also increase the power to detect collective moderate risk associations and can allows for evaluation of more biologically relevant and interpretable genetic effects particularly with genetically complex disorders (Juraeva et al., 2014). In this study we identified pathway-specific miR-137 target gene sets, and evaluated their risk association both within the PGC Stage 1 GWAS data (Ripke et al., 2011) and within a smaller independent dataset including subjects from the Mind Clinical Imaging Consortium (MCIC) (Gollub et al., 2013) and Northwestern University (NU) (Wang et al., 2013). The evaluation of pathway-specific gene sets in this manner allows for an estimation of schizophrenia-risk due to miR-137 dysregulation.

## Materials and methods

### miR-137 regulated gene curation and prediction

Experimentally validated targets and 2 indirectly regulated genes (*MAPK1* and *MAPK3*) were curated (36 in total) from the literature as described previously for the identification of 26 regulated genes in Wright et al. (2013). Additionally *HTT* (Kozlowska et al., 2013), *TBX3* (Jiang et al., 2013), *GLIPR1* (*RTVP-1*) (Ariel Bier et al., 2013), *CLDN11, GABRA1, NRXN1, NEFL, ZNF365, NECAP1*, and *RAPGEF5* (Boudreau et al., 2014) were included as validation experiments were published since (Wright et al., 2013). Targets were predicted using TargetScan version 6.2, released June 2012 (Lewis et al., 2005). Target prediction databases are known to include false positive miRNA-mRNA interactions and to exclude true interactions (Zheng et al., 2013). TargetScan offers two scoring systems to improve confidence in target-miRNA prediction: the probability of conserved targeting (Pct) score and the context score. The Pct score (with a range from 0 to 1, with 1 indicating highest) is derived by evaluating the conservation of the interaction site sequence across species (Friedman et al., 2009). Highly conserved binding sites are more likely to be functionally relevant and effective in inducing subsequent mRNA repression (Nielsen et al., 2007; Friedman et al., 2009). However, target interactions that may have evolved later in primates and humans are less likely to be conserved (Glazov et al., 2008; Friedman et al., 2009) and may be more relevant to higher order cognition and complex behavior phenotypes, such as those affected in schizophrenia. Thus some human-specific or primate-specific targets may be lost based on conservation score (Farh et al., 2005; Grimson et al., 2007). The context score provides confidence for the less conserved targets and improved confidence for conserved targets. This score (with a range from 0 to −1, with −1 indicating more probable binding) is based on evaluation of site efficacy including seed site interaction type, nearby nucleotides, site location, and seed site interaction stability among other criteria (Grimson et al., 2007; Garcia et al., 2011). Therefore, site conservation and site efficacy were both used, either separately or combined, to better capture the potential impact of miR-137 on biological pathways.

The four predicted miR-137 target gene lists, each including validated targets, that were curated for further analysis (Supplemental Table 1) included: (a) the full target list as predicted by TargetScan (full target list), (b) targets with Pct scores greater than or equal to 0.9 (conserved target list), (c) targets with the best 50% of context scores (context target list), and (d) the high Pct and low context scoring targets (intersection target list). The Pct score cutoff of 0.9 was based on previous work of Ripke et al. (2011). The context score, with an equal percentage of predicted targets as that chosen for the Pct score, was −0.12. Finally, the intersection of targets with Pct scores greater than or equal to 0.9 and with context scores below −0.12 was used as a higher confidence predicted list that represents more plausible conserved targets. The full lists of these gene sets are shown in Supplementary Table 2 (see yellow highlighted cells).

### Pathway selection criterion

Selection of gene sets was based on prior pathway analysis of the full list of TargetScan predicted targets and validated targets using Ingenuity Pathway Analysis (IPA) as described previously in Wright et al. (2013). From this analysis it was determined that several possibly schizophrenia-relevant pathways were significantly enriched with potential miR-137 target genes. The top 10 enriched pathways for potential targets, listed in Table 1 were selected for pathway-specific gene set enrichment analyses. Since IPA now allows for multiple testing correction of pathway enrichment, this was reassessed with the Benjamini-Hochberg multiple testing correction and all pathways were still found to be among the top 11 significantly enriched pathways (corrected *p* < 0.01) (Supplemental Table 3). Gene sets of miR-137 target genes of varied prediction confidence were created for each pathway using the target gene lists described above. See Supplemental Table 2 for the full lists of genes within each tested gene set.

**Table 1:** **Gene sets of potential hsa-miR-137 targets evaluated in MAGENTA**.

**Evaluated gene set in MAGENTA**	**Full list**	**Conserved list**	**Context list**	**Intersection list**	**Validated list**
Gene Set size	1154	560	597	311	36
**PATHWAY SPECIFIC GENE SETS**					
Sertoli cell junction signaling	27	16	15	11	4
Mechanisms of cancer	40	25	22	17	8
Hepatocyte growth factor (HGF) signaling	18	13	12	9	6
Ephrin receptor signaling	25	16	12	10	3^a^
Agrin interactions at neuromuscular junctions	14	9	8	6	3^a^
Gonadotropin Releasing Hormone (GNRH) signaling	20	12	6	6	3^a^
Cardiac –B adrenergic signaling	20	10	4	2	1
Synaptic long term potentiation (LTP)	19	11	7	4	3
Protein kinase A (PKA) signaling	42	26	18	10	4
Axonal guidance signaling	42	20	18	13	3^a^

*This table shows the number of potential miR-137 target genes evaluated in each gene list and the number of genes in each pathway-specific gene set derived from each target gene list. MAGENTA, Meta Gene Set Enrichment of Variant Analysis*.

a *Identical gene sets*.

### Meta gene set enrichment of variant analysis (MAGENTA)

The MAGENTA software program (Segrè et al., 2010) evaluates enrichment of modest associations with a disease or trait within gene sets using GWAS disease association *p*-values and odds ratios. MAGENTA includes SNPs within a region from 110 kb upstream to 40 kb downstream of each gene's transcript boundaries. The SNP with the smallest disease association *p*-value within this region is determined for later analysis as the “gene's best” association *p*-value. Such a procedure helps overcome the “watering-down” effects that occur when analyzing the average SNP *p*-value across a gene, where unassociated SNPs can depreciate gene association. The following confounds are addressed by correcting the smallest gene SNP *p*-values with step-wise linear regression: gene size, number of SNPs per gene kb, number of independent SNPs per gene kb, number of recombination spots per gene kb, linkage disequilibrium units per gene kb, and genetic distance per gene kb (Segrè et al., 2010).

MAGENTA uses corrected best gene disease association *p*-values to evaluate the enrichment of each gene set with, in this case, genes containing a schizophrenia-associated variant. Gene sets are compared to 10,000 random gene sets of identical size. The gene set *p*-value is calculated as the fraction of random gene sets with a sum rank *p*-value equal or smaller than that of the tested gene set. Gene sets with a one-tailed Mann-Whitney like rank-sum based false discovery rate (FDR) (Sabatti et al., 2003) *q*-value of <0.05 were considered significantly enriched with associated SNPs based on the FDR gene score enrichment cutoff of 75%. This cutoff is based on the fraction of *p*-values lower than 75% of all gene *p*-values and is suggested for polygenic diseases such as is proposed for schizophrenia (Purcell et al., 2009) where association values may be more modest (Segrè et al., 2010).

### Database and GWAS information

To determine whether the gene sets in Table 1 are enriched in schizophrenia risk variants, *p*-values from two independent GWAS were evaluated. The first *p*-values were derived from the stage 1 GWAS study reported in Ripke et al. (2011), the GWAS in which the schizophrenia risk association was discovered for the miR-137 host gene SNP, rs1625579. This analysis included 21,856 subjects (9394 cases and 12,462 controls) of European ancestry from the Psychiatric GWAS Consortium (PGC) (Supplemental Table 4). Genotyping was performed using Affymetrix and Illumina Chips across samples. Quality control was conducted as described in Ripke et al. (2011). The unadjusted *p*-values and odds ratios for the 1.2 million SNPs evaluated in this GWAS are available on the PGC website, http://www.med.unc.edu/pgc/downloads. GWAS *p*-values and odds ratios were loaded into MAGENTA (Segrè et al., 2010) with one gene set file including all gene sets in Table 1 to allow cross-comparison of gene sets, as permutation differences can cause slightly different results.

To evaluate if the PGC MAGENTA results were replicable, subjects were analyzed from the Mind Clinical Imaging Consortium (MCIC) (Gollub et al., 2013) and Northwestern University (NU) (Wang et al., 2013) (Supplemental Table 4). All participants provided written informed consent, and the Institutional Review Board at each site approved this project. Genotyping was conducted at the Mind Research Network Neurogenetics Core lab using Illumina HumanOmni-Quad 1M and 5M BeadChips respectively. Only Caucasian subjects were used to avoid population-specific effects. Caucasians were identified using the Enhancing Neuroimaging Genetics through Meta-Analysis (ENIGMA) multi-dimensional scaling (MDS) protocol within the imputation protocol (http://enigma.ini.usc.edu/protocols/genetics-protocols/). Genotype data from each dataset was merged using PLINK after updating the MCIC SNP locations to match that of the more recent NU data. Quality control was performed before and after merging similarly to that of Ripke et al. (2011), using PLINK with the following thresholds: Hardy Weinberg equilibrium *P* < 10^−6^, minor allele frequency < 0.05, missing rate per SNP < 0.02, missing rate per individual < 0.02. Relatedness and population stratification testing was performed using PLINK. Outliers were identified and removed as well as one individual per pair that appeared to be related, according to pi-hat values of 0.05 or greater per pair of individuals. After all quality control and pruning, a total of 244 individuals remained (103 cases and 141 controls) and 539,288 SNPs.

A GWAS study using logistic regression covarying for chip type was performed on the merged MCIC and NU genotypic data with a genomic inflation factor of 1.00737. The *p*-values obtained were evaluated using MAGENTA for the previously significantly enriched gene sets found in the PGC data.

## Results

### Magenta analyses of miR-137 predicted and validated target lists

**Figure 1:**
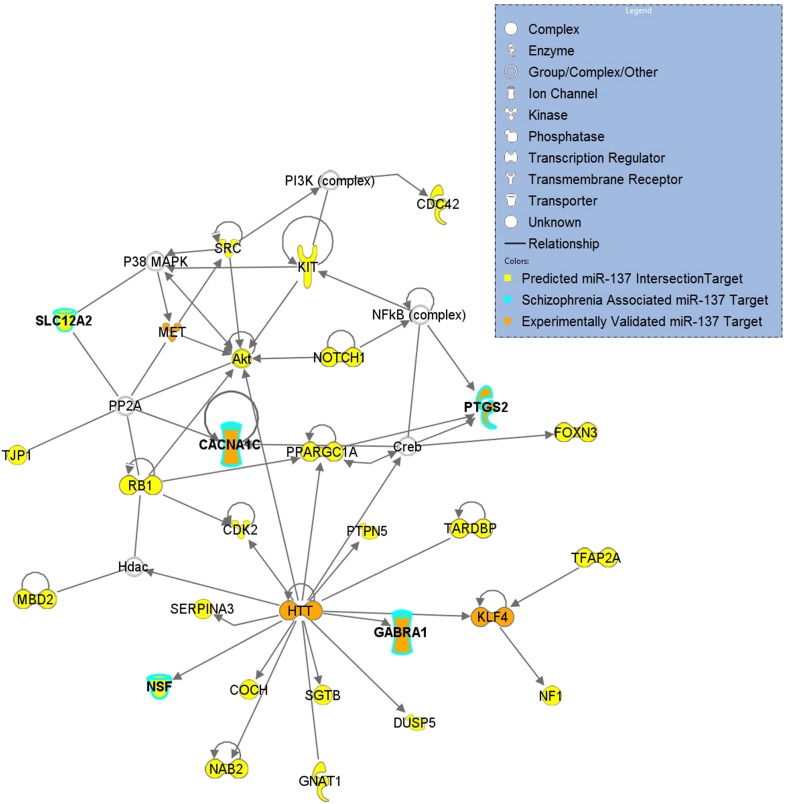
**miR-137 Intersection Target Network of Neurological Disease**. Top network derived from a core analysis using Ingenuity Pathway Analysis with the 77 genes of the intersection target list that are associated with Neurological Disease. Predicted targets are indicated in yellow, targets associated with schizophrenia are outlined in light blue and validated targets are indicated in orange.

Gene sets for each of the target lists of different prediction confidences were first evaluated for enrichment of schizophrenia risk SNPs (Table 2). The higher confidence predicted lists, i.e., the conserved, context, and intersection, were all significantly enriched with schizophrenia-associated variants. As shown in Figure 1, the IPA network derived from the 77 intersection targets that were associated with neurological disease contains many nervous system expressed genes including some schizophrenia-associated genes that interact with one another. The validated target list was not significantly enriched with variants, likely due to the small size of this set. The full list of putative targets, although trending, was not enriched either, possibly due to a higher inclusion of false positive miRNA-target interactions. This suggests that the *high confidence predicted miR-137 target genes* overall contain SNPs that are associated with schizophrenia.

**Table 2:** **Curated hsa-miR-137 target gene lists show enrichment for association with schizophrenia**.

**Gene set**	**MAGENTA Gene set size**	**Nominal GSEA *p*-value with 75% cutoff**	**FDR *q*-value with 75% cutoff**
Full List	1061	1.32E-02	5.11E-02
**Conserved List**	548	1.50E-03	**1.96E-02**
**Context List**	585	3.90E-03	**1.89E-02**
**Intersection List**	329	2.50E-03	**1.90E-02**
Validated List	36	9.08E-02	9.75-02

*This table shows the results of the target gene lists including the number of genes after conversion to ENTREZ IDs, the gene set enrichment analysis (GSEA) p-value before false discovery rate (FDR) correction, and the FDR q-value used to determine significance at a threshold of q < 0.05. Significant gene lists are shown in bold. MAGENTA, Meta Gene Set Enrichment of Variant Analysis*.

### MAGENTA analyses of miR-137 target pathway gene sets

The schizophrenia-risk of miR-137 validated and predicted targets was assessed after targets were classified within canonical pathways according to IPA. Meta gene set enrichment of variant association analysis using MAGENTA software (Segrè et al., 2010) revealed several pathway relevant gene sets of miR-137 targets significantly enriched with schizophrenia-associated variants (Table 3). For a list of the variants identified for each significant gene set see Supplementary Table 5. Ephrin receptor signaling, axonal guidance signaling, and Sertoli cell junction signaling gene sets were significantly enriched with schizophrenia-associated variants from four out of five gene lists (Table 3). The enrichment found for nearly all gene sets specific to these pathways using higher confidence target lists, strongly suggests that these pathways are indeed enriched with miR-137 target genes associated with schizophrenia risk. Additionally, synaptic LTP gene sets from the intersection and validated gene lists, and PKA signaling gene sets from the full and validated target lists, were enriched in risk genes. The mechanism of cancer gene set was also significantly enriched, but only from the validated target list. Overall, analysis of pathway specific gene sets derived from the multiple potential target lists provided higher confidence for the potential impact of this miRNA within these pathways.

**Table 3:** **Significantly enriched hsa-miR-137 pathway-specific gene sets**.

**Gene set**	**Gene list**	**MAGENTA gene Set size**	**Nominal GSEA *p*-value with 75% cutoff**	**FDR *q*-value with 75% cutoff**
Axonal guidance signaling	Conserved	19	8.00E-04	4.85E-03
	Context	18	5.60E-03	1.99E-02
	Intersection	13	5.80E-03	2.13E-02
	Full	40	1.14E-02	2.82E-02
Ephrin receptor signaling	Conserved	16	4.10E-05	7.00E-04
	Full	25	1.00E-03	8.43E-03
	Context	12	2.20E-03	1.18E-02
	Intersection	10	3.80E-03	1.36E-02
Synaptic LTP	Validated	3	1.69E-02	1.89E-02
	Conserved	13	2.19E-02	3.23E-02
Mechanisms of Cancer	Validated	8	2.59E-02	2.75E-02
PKA signaling	Full	46	1.03E-02	2.97E-02
	Validated	4	4.73E-02	4.18E-02
Sertoli cell junction signaling	Intersection	11	7.40E-03	1.84E-02
	Conserved	16	6.40E-03	1.84E-02
	Context	15	1.48E-02	2.75E-02
	Validated	4	5.24E-02	4.88E-02

*This table shows the results for the pathway-specific gene sets of able 1 including the number of genes after conversion to ENTREZ IDs, the gene set enrichment analysis (GSEA) p-value before false discovery rate (FDR) correction, and the FDR q-value used to determine significance at a threshold of q < 0.05. All gene sets pass the significance threshold. MAGENTA, Meta Gene Set Enrichment of Variant Analysis*.

### MCIC and NU replication cohort results

**Figure 2:**
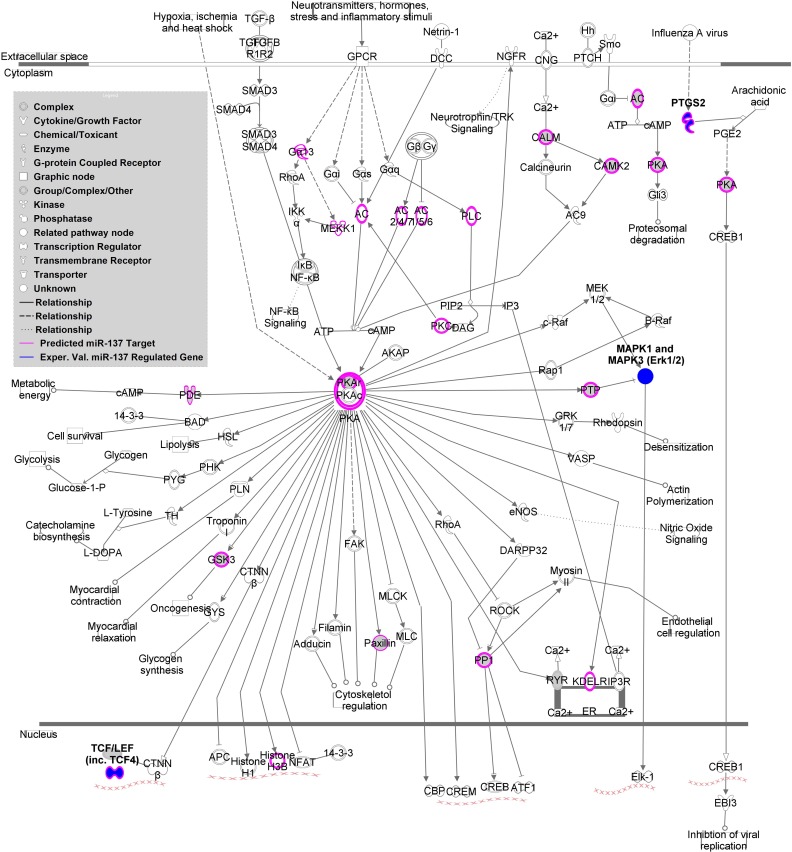
**miR-137 targets within Protein Kinase A (PKA) Signaling Pathway**. This figure depicts the canonical PKA signaling pathway according to Ingenuity Pathway Analysis. Predicted miR-137 targets based on TargetScan are indicated in pink and experimentally validated regulated genes are indicated in dark blue.

MAGENTA analysis of a replication cohort using the MCIC (Gollub et al., 2013) and NU (Wang et al., 2013) dataset GWAS association *p*-values revealed one significantly enriched gene set. The PKA signaling gene set from the validated target list, including *TCF4* and *PTGS2*, as well as *MAPK1, MAPK3* (experimentally validated indirectly regulated genes), was significantly enriched with a nominal GSEA *p*-value of 0.003 and an FDR *q*-value of 0.014. Supplemental Table 6 shows the top SNPs from this analysis. As shown in Figure 2, the canonical PKA signaling pathway from IPA is enriched with predicted and validated miR-137 regulated genes, suggesting that this pathway may be involved in the mechanism of the miRNA in schizophrenia.

## Discussion

Current research about the first discovered *MIR137* host gene SNP in schizophrenia, rs1625579, suggests that this variant impacts some endophenotypic measures in patients and controls in a different manner (Whalley et al., 2012; Lett et al., 2013). In addition to the SNPs in this same locus discovered in the Ripke et al. (2013, 2014), variable number tandom repeats (VNTRs) and rare variants (Duan et al., 2014; Strazisar et al., 2014; Warburton et al., 2014) within the locus may also influence schizophrenia risk. miR-137 has been shown to alter the expression of genes involved in neuronal differentiation (Hill et al., 2014) and to downregulate genes associated with schizophrenia risk (Collins et al., 2014). Therefore, it is likely that the interaction of these schizophrenia-associated *MIR137* variants with additional variants within miR-137 regulated genes present in patients may increase both dysregulation by this miRNA and schizophrenia risk. These additional variants may disrupt targeting efficiency or lead to general disruption of pathway-specific genes, collectively altering biological processes required for proper brain functioning.

We have previously shown that miR-137 target genes fall within certain biological pathways more frequently than expected by chance (Wright et al., 2013). Data presented in this study clearly indicates that the genes in these miR-137 regulated pathways are also enriched with schizophrenia risk variants, suggesting a potential mechanism for the association of the *MIR137* risk variant. These pathways include Ephrin receptor signaling targets, axonal guidance signaling targets, synaptic LTP targets, Sertoli cell junction signaling targets, and PKA signaling targets. As described below, there is supporting evidence for how each of these may indeed enhance the risk of schizophrenia or impact the etiology of the disorder.

Ephrin receptor signaling is closely linked to axonal guidance and synaptic LTP, particularly NMDA dependent plasticity (Filosa et al., 2009). Interactions of the Ephrin receptors across adjacent neurons or glial cells and neurons help to guide axonal repulsion (Xu and Henkemeyer, 2012), dendritic spine stability, synaptogenesis (Lin and Koleske, 2010), and control synapse glutamate concentrations (Filosa et al., 2009), all of which impact LTP. Schizophrenia imaging genetics studies have found associations between axonal guidance signaling genes and prediction of fMRI measures of dorsolateral prefrontal cortex (DLPFC) inefficiency during a working memory task (Walton et al., 2013). A link between altered LTP and schizophrenia was shown more directly in a transcranial direct current stimulation (tDCS) study, which found altered LTP-like plasticity in patients (Hasan et al., 2013). Additionally, schizophrenia animal models using NMDAR antagonists, have shown effects on both LTP and behavior measures demonstrating similar alterations to the cognitive, negative, and positive symptoms found in humans (Wiescholleck and Manahan-Vaughan, 2013). This suggests that Ephrin receptor signaling and axonal guidance alterations leading to changes in NMDA driven synaptic LTP alterations could lead to all three spectra of symptoms associated with the disorder.

The enrichment of Sertoli cell junction signaling gene sets is compelling as increased risk of schizophrenia is associated with increased paternal and grandpaternal age (Frans et al., 2011). Sertoli cells create the supportive niche for the spermatogonial stem cells and create the blood-testis barrier (Kaur et al., 2014). It is suggested that reduced Sertoli cell population with age may reduce both germ cell production and quality (Paul and Robaire, 2013). Perhaps alterations in Sertoli cell function via dysregulation by this miRNA could also reduce germ cell quality of patient fathers and grandfathers.

Finally, the miR-137 validated target PKA signaling gene set (*MAPK1, MAPK3, TCF4*, and *PTGS2*) is of particular interest given that enrichment of schizophrenia-risk associated variants within these targets was replicated in an independent cohort. PKA is involved in the biological pathways of two neurotransmitters implicated schizophrenia, as it not only modulates glutamate signaling but also responds to dopamine signaling (Sarantis et al., 2009). PKA signaling also appears to play a critical role in the synergistic interactions between these two neurotransmitter signaling cascades within the hippocampus and prefrontal cortex through activity of MAPK1 and MAPK3 (ERK1/2) (Sarantis et al., 2009).

PKA signaling is also critical for maturation of prefrontal cortex D1 excitability in adolescence, a region well-known for alterations in schizophrenia and a time period of particular vulnerability (Heng et al., 2011). Inhibition of phosphodiesterase 4 (PDE4), an enzyme implicated in schizophrenia and involved in the auto-inhibition of PKA signaling, increases D1 signaling in pyramidal neurons of the prefrontal cortex and enhanced sensory gating behavior in mice as measured by prepulse inhibition (PPI) (Juraeva et al., 2014). Interestingly, both schizophrenia and control subjects carrying a schizophrenia-risk associated variant within the *TCF4* gene and mice moderately overexpressing TCF4, a transcription factor downstream of PKA signaling, (Figure 2) also have disrupted PPI activity (Brzózka et al., 2010 and Quednow et al., 2014). Evidence for a role of this molecule in schizophrenia is extensive (Quednow et al., 2014). *TCF4* mRNA expression is increased in human induced pluripotent stem cells (hiPSC) from schizophrenia patients (Brennand et al., 2011) and increased in postmortem DLPFC samples of miR-137 risk SNP carriers (Guella et al., 2013).

The remaining PKA gene set molecule, *PTGS2*, encoding the COX-2 protein, is gaining attention as a schizophrenia drug target because inhibitors appear to be beneficial in symptom treatment (Müller et al., 2010; Baheti et al., 2013). *PTGS2* mRNA expression is altered in the prefrontal cortex of patients (Tang et al., 2012). This risk gene is relevant to the inflammatory basis theories for schizophrenia (Feigenson et al., 2014). As depicted in Figure 2, *PTGS2* is involved in inflammatory processes such as infection, a potential risk factor for the disorder.

New research suggests that the standard methods for functional enrichment analyses of miRNA regulated genes may be biased and inaccurate, resulting in false positive findings. This bias is due to a lack of multiple testing correction and a high rate of false positives in miRNA target prediction (Bleazard et al., 2015). To assess the validity of our pathway selection, we reanalyzed the pathway enrichment of our predicted and validated miR-137 regulated genes and found that these pathways were still significantly enriched following multiple testing correction (corrected *p* < 0.01). We then limited our list of predicted miR-137 regulated genes to only those with CLIP-Seq evidence in the starBase v2.0 database (Yang et al., 2011; Li et al., 2014) and again found all pathways to be significantly enriched (corrected *p* < 0.05). Additionally we did a final evaluation irrespective of TargetScan prediction of only the miR-137 regulated genes with at least five supporting experiments in the starBase database and, and again found that all pathways except synaptic LTP and cardiac B-adrenergic signaling were among the most significantly enriched following multiple testing correction (*p* < 0.05). These results (Supplemental Table 3) strongly suggest that our pathway selection was valid and that these pathways may be especially vulnerable to alterations in miR-137 regulation. Furthermore, our effort to evaluate gene sets derived from a variety of higher-confidence target lists help verify our schizophrenia-risk enrichment findings by reducing the inclusion of falsely predicted miR-137 targets, while still evaluating true targets that may be eliminated by higher prediction constraints. Still, there is the potential that our lists include a few falsely predicted target genes and are missing some true targets not predicted by TargetScan. Our inclusion of experimentally validated miR-137 regulated genes assists with this, but this list is still limited to a small subset of targets.

Another limitation of this analysis is the lack of evaluation for the possible creation of new binding sites from polymorphisms in unpredicted target genes. Our current tools do not allow evaluation of how target SNPs might impact regulation. Given the heterogeneity and polygenicity of schizophrenia, our replication sample size was too small to allow full replication of many of our results (Purcell et al., 2009). However the replication of associated variants within the validated target PKA signaling gene set suggests that other signaling pathways gene set risk association may be replicated in a larger sample and greatly strengthens our findings for this particular gene set. Moreover, the use of the PGC stage 1 dataset (Ripke et al., 2011) provided a unique opportunity for use of a very large dataset giving confidence to our findings. Further replication with larger sample sizes will help validate our results.

Despite these limitations, our analysis of the enrichment of schizophrenia-associated variants within pathway specific gene sets of potential miR-137 targets suggests that these pathways are particularly vulnerable to dysregulation by this miRNA. Further research to evaluate the influence of this miRNA on these pathways in schizophrenia is therefore warranted.

## Conclusions

Genetic association studies indicate that variants within miRNAs and targets can have great impact on specific diseases (Abelson, 2005; Wang et al., 2008). These studies often evaluate variants within one risk gene of interest at a time, and discover alterations in miRNA binding to that specific target risk gene. However, each miRNA has the capacity to target hundreds of targets and impact many different pathways, so determining possible variants associated with disease that impact miRNA regulation, can be challenging. Thus studies like this, evaluating many putative and validated targets, are necessary first steps to guide further research on the impact of specific miRNAs in diseases.

Many schizophrenia relevant pathways were previously identified to have an overrepresentation of miR-137 target genes. Our findings of schizophrenia-associated variants within PKA signaling and other pathways provide a map to guide further investigation of the role of this miRNA in this illness.

## Author contributions

CW helped design the study, carried out the analyses, and drafted the manuscript. NPB and JT designed the study and assisted in interpreting the data and writing the manuscript. VC, LW, and SE revised the manuscript and assisted in data interpretation. All authors have read and approved the final manuscript.

### Conflict of interest statement

The authors declare that the research was conducted in the absence of any commercial or financial relationships that could be construed as a potential conflict of interest.
